# Supplementary material for: Exposure to heat stress leads to striking clone-specific nymph deformity in pea aphid
Source: PLoS One. 2023 Oct 26;18(10):e0282449. doi: 10.1371/journal.pone.0282449 (PMC10602343; doi:10.1371/journal.pone.0282449)
Supplement: S1 File — List of reference genes tested to check expression stability; S2 Table. Details of two candidate genes (HSP70 and HSP83) used in qPCR; S3 Table. Analysis of the total number of nymphs (live and deformed); S4 Table. Total plant dry weight (TDW); S5 Table. Analysis of HSP70 expression; S6 Table. Posthoc test of HSP70 expression; S7 Table. Analysis of HSP83 expression]. (DOCX) [file pone.0282449.s001.docx]

Supporting information

**Exposure to heat stress leads to striking clone-specific nymph deformity in pea aphid**

Hawa Jahan ^1,2*¶^, Mouhammad Shadi Khudr ^1¶^, Ali Arafeh ^3^, Reinmar Hager ^1^

^1^ Division of Evolution, Infection and Genomics, School of Biological Sciences, Faculty of Biology, Medicine and Health, Manchester Academic Health Science Centre, The University of Manchester, Manchester, M13 9PT, UK

^2^ Department of Zoology, Faculty of Biological Sciences, University of Dhaka, Dhaka – 1000, Bangladesh

^3^ Chemical Engineering, Faculty of Science and Engineering, James Chadwick Building, The University of Manchester, Manchester, M13 9SS, UK

* Corresponding author:

Email: hawa.jahan@postgrad.manchester.ac.uk

**^¶^**These authors contributed equally to this work.

**Running Title:** Striking pea aphid nymph deformity

**Declarations of interest:** none

***Authorship contribution statement***

Hawa Jahan (HJ): Conceptualisation, methodology, investigation, formal analysis, data curation and visualisation, resources, writing (original draft, review, and editing), funding acquisition, co-produced the manuscript with MSK.

Mouhammad Shadi Khudr (MSK): Conceptualisation, methodology, project administration, formal analysis and visualisation, writing (original draft, review, and editing), co-produced the manuscript with HJ.

Ali Arafeh (AA): Formal analysis, visualisation, writing (review).

Reinmar Hager (RH): Methodology, writing (review, and editing), resources, project administration, supervision.


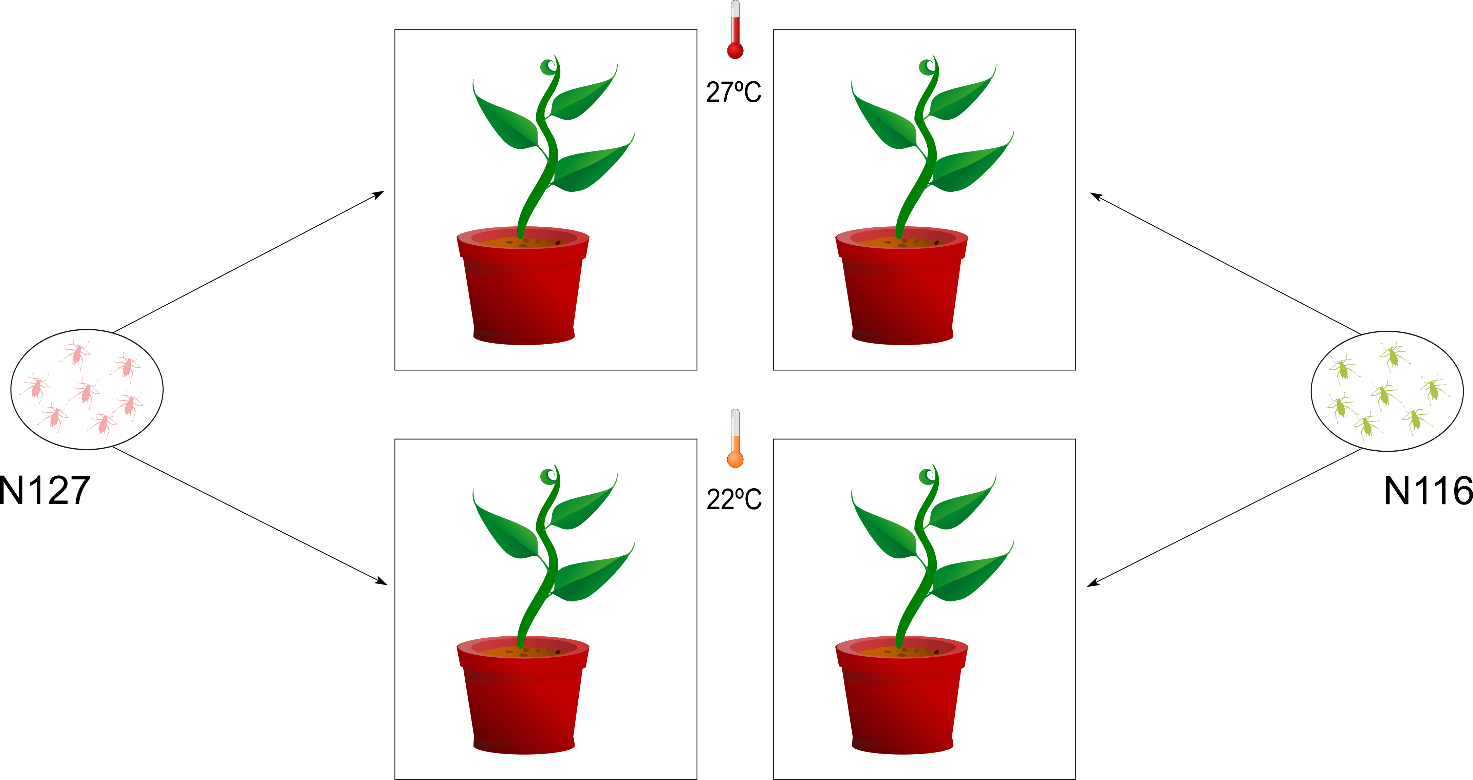


**Fig S1. Experimental design.** Seven first instars of each aphid clone (N127 [pink] or N116 [green]) were introduced to 2-week-old plants of one cultivar of fava bean grown and maintained at 22°C (thermal optimum) or 27°C (constant heatwave as thermal stress). Aphids were reared in the respective conditions for 14 days where they developed and reproduced. On day 15, aphid total numbers per enclosure (n=60) were counted and samples were taken for further analyses. Plants were washed at the end of the experiment and dried out in an oven at 55°C for two nights.

*The information shown in (S1 Table) is based on the work done by Yang et al. (2014) [1].*

**S1 Table. List of reference genes tested to check expression stability.** The table shows five reference genes that were randomly selected from a list of ten common genes in pea aphid under different temperature conditions where the genes showed higher stability. See main text Methods for further details.

| Gene name | Accession no | Primer sequence | Length (bp) |
| --- | --- | --- | --- |
| Succinate dehydrogenase B (SDHB) | NM_001162436 | CTGAATTCCTGTGGACCTATGG | 90 |
|  |  | ACGGCAAGAACGCCTAAA |  |
| 16S ribosomal RNA (16S) | FJ411411 | AGAAACCAACCTGGCTTACAC | 121 |
|  |  | TTGCGACCTCGATGTTGAATTA |  |
| NADH dehydrogenase (NADH) | NM_001162323 | CGAGGAGAACATGCTCTTAGAC | 93 |
|  |  | GATAGCTTGGGCTGGACATATAG |  |
| Elongation factor 1 alpha (EF1A) | AY219737 | AGAATGGACAAACCCGTGAA | 104 |
|  |  | GCTGTATGGTGGTTCAGTAGAG |  |
| Ribosomal protein L12 (RPL12) | NM_001162323 | AAGGCTACATCTGACTGGAAAG | 101 |
|  |  | ACCAATGATGATGCAGAAGGA |  |

**S2 Table.** **Details of two candidate genes (HSP70 and HSP83) used in qPCR.** The table shows the name, function, primer sequence, and product size of the HSP genes that were used in the qPCR analysis specified in the main text Methods. The differential expression levels of these genes were tested in two pea aphid clones (N127 and N116) under thermal stress (27°C) compared to thermal optimum (22°C). See main text Methods for further details.

| Gene name | Transcript ID | Gene function | Primer sequence | Length (bp) |
| --- | --- | --- | --- | --- |
| Heat shock protein 70 A1-like (HSP70) | XM_001945751.5 | Carbohydrate metabolic process | TAGAGGGCCTACCGTCGAAGA | 190 |
|  |  |  | GTGAAAACTTGGGTGCTTACAC |  |
| Heat shock protein 83 (HSP83) | XM_001943137.5 | Response to stress | ACACTGTTTGCCGGTTGCAG | 147 |
|  |  |  | CGGCTTGGAAAGCGAAGGTC |  |

**S3 Table.** **Analysis of the total number of nymphs (live and deformed).** The table shows the outcome of the Manova (Type II) model testing the total number of nymphs produced by the survived mothers. The predictors were (i) Thermal stress (two levels: 22°C [thermal optimum, control], 27°C [thermal stress]), (ii) Aphid clone (N116 [green], N127 [pink]), (iii) plant total dry weight (TDW) as a covariate, and (iv) the interactions of these predictors. The binary response variable was (deformed nymphs [DJ], live nymphs [LJ]). See main text Methods for further details. Significant results are shown in bold. TS = thermal stress, Aphid C = Aphid clone, Df = Degrees of freedom, Pillai = Pillai's trace (a test statistic produced by MANOVA), approx F = approximated F, Num Df = Number of degrees of freedom, Den Df = Number of degrees of freedom associated with the model errors, Pr(>F) = p-value associated with the F statistic.

|  | Df | Pillai | approx F | num Df | den Df | Pr(>F) |
| --- | --- | --- | --- | --- | --- | --- |
| TDW | 1 | 0.14 | 4.012 | 2 | 51 | **0.024** |
| TS | 1 | 0.90 | 234.76 | 2 | 51 | **<0.0001** |
| Aphid C | 1 | 0.43 | 19.04 | 2 | 51 | **<0.0001** |
| TDW X TS | 1 | 0.004 | 0.11 | 2 | 51 | 0.896 |
| TDW X Aphid C | 1 | 0.09 | 2.49 | 2 | 51 | 0.093 |
| TS X Aphid C | 1 | 0.42 | 18.31 | 2 | 51 | **<0.0001** |
| TDW X TS X Aphid C | 1 | 0.13 | 3.71 | 2 | 51 | **0.031** |
| Residuals | 52 |  |  |  |  |  |

**S4 Table.** **Total plant dry weight (TDW).** Means (± SE), standard deviation (SD), and relative standard deviation (RSD) of the total dry weight of the fava bean host plant. There were 60 enclosures in total including 2 aphid clones X 2 thermal conditions [control (favourable conditions at 22˚C or stress at 27˚C)] X 15 replicates. See main text Methods for further details.

| Clone | Thermal regime | Mean | SD | SE | RSD |
| --- | --- | --- | --- | --- | --- |
| N127 | Thermal optimum | 46.60 | 8.07 | 2.08 | 17.32 |
| N127 | Thermal stress | 44.07 | 7.83 | 2.02 | 17.76 |
| N116 | Thermal optimum | 40.33 | 7.38 | 1.91 | 18.30 |
| N116 | Thermal stress | 45.27 | 11.53 | 2.98 | 25.46 |

*S5-S7 Tables, shown below, provide the outcome of the analysis of the expression of the HSP genes from an alternative perspective to that adopted in the main text. Here, the statistical analyses of the expression of HSP70 and HSP83 are done individually.*

**S5 Table.** **Analysis of HSP70 expression.** The table shows the outcome of the Anova (Type II) model testing changes in the expression of the HSP70 gene in two pea aphid clones (N127 and N116) under thermal stress (27°C) compared to thermal optimum (22°C). See main text Methods for further details. Significant results are shown in bold, Sum Sq = Sum of Squares.

|  | Sum Sq | Df | F value | Pr(>F) |
| --- | --- | --- | --- | --- |
| Thermal stress | 0.22 | 1 | 3.12 | **0.115** |
| Aphid clone | 1.72 | 1 | 24.85 | **0.001** |
| Thermal stress X Aphid clone | 1.37 | 1 | 19.84 | **0.002** |
| Residuals | 0.55 | 8 |  |  |

**S6 Table.** **Posthoc test of HSP70 expression.** The table shows the outcome of the posthoc TUKEY test following the Anova (Type II) model testing changes in the expression of the HSP70 gene in two pea aphid clones (N127 and N116) under thermal stress (27°C) compared to thermal optimum (22°C). See main text Methods for further details. Only significant results are shown.

|  | estimate | SE | df | t.ratio | p.value |
| --- | --- | --- | --- | --- | --- |
| N127‒control *vs.* N127‒severe | -1.43 | 0.22 | 8 | -6.68 | **0.001** |
| N116‒severe *vs.* N127‒severe | -0.94 | 0.22 | 8 | -4.4 | **0.01** |

**S7 Table.** **Analysis of HSP83 expression.** The table shows the outcome of the Anova (Type II) model testing changes in the expression of the HSP83 gene in two pea aphid clones (N127 and N116) under thermal stress (27°C) compared to thermal optimum (22°C). See main text Methods for further details. Significant results are shown in bold.

|  | Sum Sq | Df | F value | Pr(>F) |
| --- | --- | --- | --- | --- |
| Thermal stress | 0.49 | 1 | 5.6 | **0.046** |
| Aphid clone | 0.16 | 1 | 1.82 | 0.214 |
| Thermal stress X Aphid clone | 0.0003 | 1 | 0.003 | 0.952 |
| Residuals | 0.70 | 8 |  |  |

**References**

[1] Yang C, Pan H, Liu Y, Zhou X. Selection of reference genes for expression analysis using quantitative real-time PCR in the pea aphid, *Acyrthosiphon pisum* (Harris) (Hemiptera, Aphidiae). *PLoS ONE.* 2014; 9: e110454. doi: 10.1371/journal.pone.0110454
